# Rapid anti‐depressant‐like effects of ketamine and other candidates: Molecular and cellular mechanisms

**DOI:** 10.1111/cpr.12804

**Published:** 2020-04-07

**Authors:** Fan Zhen Peng, Jie Fan, Tong Tong Ge, Qian Qian Liu, Bing Jin Li

**Affiliations:** ^1^ Jilin Provincial Key Laboratory on Molecular and Chemical Genetics The Second Hospital of Jilin University Changchun China

**Keywords:** depression, ketamine, neural circuit, rapid anti‐depressant, synaptic plasticity

## Abstract

Major depressive disorder takes at least 3 weeks for clinical anti‐depressants, such as serotonin selective reuptake inhibitors, to take effect, and only one‐third of patients remit. Ketamine, a kind of anaesthetic, can alleviate symptoms of major depressive disorder patients in a short time and is reported to be effective to treatment‐resistant depression patients. The rapid and strong anti‐depressant‐like effects of ketamine cause wide concern. In addition to ketamine, caloric restriction and sleep deprivation also elicit similar rapid anti‐depressant‐like effects. However, mechanisms about the rapid anti‐depressant‐like effects remain unclear. Elucidating the mechanisms of rapid anti‐depressant effects is the key to finding new therapeutic targets and developing therapeutic patterns. Therefore, in this review we summarize potential molecular and cellular mechanisms of rapid anti‐depressant‐like effects based on the pre‐clinical and clinical evidence, trying to provide new insight into future therapy.

## 1 INTRODUCTION

Major depressive disorder (MDD) is a mental disorder associated with mood disorders, characterized by depressed mood, decreased interest, cognitive impairment and even suicidal ideation. It is the main cause of global disability,^1^ and almost 20% of people will suffer one episode of depression at some point in their lifetime.^2^ Treatments of depression mainly include cognitive behavioural therapy and drug intervention. The pathogenesis of depression is associated with disorder of monoamine neurotransmitter levels. Based on the pathogenesis, drug treatments include selective serotonin reuptake inhibitors (SSRIs), serotonin and norepinephrine reuptake inhibitors (SNRIs), tricyclic anti‐depressants (TCAs) and monoamine oxidase inhibitors (MAOIs). Though traditional medications may alleviate depressive symptoms in some degree, they work slowly. It takes weeks to months for patients to benefit from drug treatment when up to 30% of those patients still do not relieve symptoms and even develop resistance after receiving medication.^3^

Unlike traditional anti‐depressants, ketamine could reduce suicidal ideation and improve mood in a short period of time^4^ (Tables 1 and 2). Ketamine is a commonly used anaesthetic and analgesic drug. Clinical study showed that intravenous injection of 0.5 mg/kg of ketamine for 40 minutes could induce a strong and rapid anti‐depressant‐like response in patients with depression,^5^ even in those who failed to treatment with traditional drugs. This effect could last 1‐2 weeks.^6^, ^7^ (R,S)‐ketamine is a racemic mixture comprising equal parts of (R)‐ketamine (arketamine) and (S)‐ketamine (esketamine). Esketamine has five times greater affinity for *N*‐methyl‐d‐aspartate receptor (NMDAR) than arketamine.^8^ Esketamine was approved by Food and Drug Administration (FDA) for adult patients with treatment‐resistant depression (TRD) in 2019. It is the first anti‐depressant in 30 years with a new mechanism. Several clinical trials demonstrated that esketamine nasal spray plus oral anti‐depressant improved symptoms.^9^ The response arose at 28 days^10^ and appeared to persist for more than 2 months.^11^ However, the clinical application of esketamine still needs to be concerned. On the one hand, the efficacy of esketamine is controversial. It was found that in the phase 3 clinical trials, the grouping criteria were not strict. About 22% of the patients only resisted to one class of drugs, which meant that they were not strictly defined TRD. Patients participated in the randomized withdrawal trial were those who had been previously randomly assigned to esketamine and achieved stable remission, leading to a statistically higher response to the drug. In addition, in the sole positive phase 3 trial, the mean decrease on the Montgomery‐Åsberg Depression Rating Scale (MADRS) was 20.8 for esketamine vs 16.8 for placebo. Besides, the result of meta‐analysis showed that the standardized mean difference (SMD) of esketamine was similar to the olanzapine‐fluoxetine combination, and less than the SMD of aripiprazole and quetiapine. These suggest that esketamine shows no significant advantage over placebo or other drugs approved by FDA. Moreover, one of the trials involved older patients and showed non‐significant results, indicating that the efficacy of esketamine in this demographic remained unclear. Finally, the rapid onset of response was not demonstrated formally. About 8%‐10% of patients who took esketamine achieved a rapid clinical response, compared with 5% of placebo. On the other hand, the results of the study 3003 were not consistent with the FDA requirement for substantial evidence of effectiveness. One site in Poland drives the overall study result due to a 100% of placebo arm relapses in this study. Removement of the outlier site changed the results from significant to non‐significant.^12^ So far, the use of esketamine has been limited to certified medical offices or clinics in America. Another isomer (R)‐ketamine is also a potential anti‐depressant which is undergoing clinical trials.^13^ It is worth noting that (R)‐ketamine has greater potency and longer‐lasting anti‐depressant effects than (S)‐ketamine in rodents.^14^, ^15^, ^16^ In fMRI test, it was shown that (R,S)‐ketamine and (S)‐ketamine significantly activated the cortex, nucleus accumbens and striatum of conscious rats, so as the NMDAR antagonist MK‐801. On the contrary, (R)‐ketamine produced negative response.^17^ Similar pattern could be observed in clinical test.^18^ These indicate that NMDAR may not be the primary target of (R)‐ketamine.^19^ (S)‐ketamine and (R)‐ketamine are also agonists of α‐amino‐3‐hydroxy‐5‐methyl‐4‐isoxazole‐propionic acid receptor (AMPAR) and both activated brain‐derived neurotrophic factor (BDNF)‐tropomyosin receptor kinase B (TrkB) pathway. It is worth noting that their mechanisms may be different. Study showed that (S)‐ketamine activated BDNF‐TrkB pathway through mTOR signalling pathway while (R)‐ketamine activated MEK‐ERK pathway, mediating the activation of BDNF‐TrkB pathway.^20^ In another study, it was shown that (R)‐ketamine could activate BDNF‐TrkB pathway and reverse the decrease in dendritic spine density, inducing synaptogenesis in the pre‐frontal cortex (PFC), CA3 and dentate gyrus (DG) of the hippocampus and eliciting sustained anti‐depressant effects in depressed rodents.^15^ Nevertheless, neither isomer attenuated the reduced BDNF in the PFC of susceptible chronic social defeat stress (CSDS) mice after 30 minutes, indicating that neither isomer improved the level of BDNF or induced synaptogenesis.^20^ Whether the long‐lasting anti‐depressant effects of (R)‐ketamine is related to MERK‐ERK signalling is unknown. Besides, detrimental side effects of (R)‐ketamine are fewer than (R,S)‐ketamine and (S)‐ketamine.^15^, ^21^ It was observed that (S)‐ketamine caused a reduction in parvalbumin (PV)‐positive cells in the medial pre‐frontal cortex (mPFC) and DG, while (R)‐ketamine did not. PV‐positive cell is related to schizophrenia, and this may be the reason why (S)‐ketamine produces psychotomimetic side effects.^15^ In addition, side effects of (S)‐ketamine are associated with mechanistic target of rapamycin (mTOR). The activation of mTOR signalling after drug abuse contributes to drug‐related behaviours such as excessive drug intake.^22^ (S)‐ketamine activates mTOR signalling in the brain regions, and this may lead to drug abuse. Moreover, a study using positron emission tomography showed that in the conscious monkey, (S)‐ketamine but not (R)‐ketamine could reduce dopamine D2/3 receptor binding in striatum.^23^ It is possible that (S)‐ketamine‐induced dopamine release relates to acute psychotomimetic side effects in humans. In addition to ketamine, other drugs^24^, ^25^, ^26^ and treatments^27^, ^28^, ^29^ can also produce rapid anti‐depressant‐like effects, but they are not long‐lasting. At present, mechanisms for the rapid anti‐depressant effects are not completely clear. Defining the mechanisms of rapid anti‐depressant‐like effects and finding pathways and targets for related drugs and physical therapies are important for developing new, safe and long‐acting therapeutic methods. Here, we highlight the potential mechanisms of rapid anti‐depressant effects.

**TABLE 1:** Summary of the rapid anti‐depressant‐like effects of ketamine in human

Patient diagnosis	Ketamine	Time (min)	Source
Major depressive disorder	0.5 mg/kg, 40‐min infusion	40	Berman^5^
Bipolar I or II depression	0.5 mg/kg, intravenous infusion	40	Zarate^138^
Treatment‐resistant depression	0.5 mg/kg, 40‐min infusion	240	Murrough^139^
Major depressive disorder	50 mg intranasal ketamine	40	Lapidus^140^
Major depressive disorder	0.5 mg/kg, 40‐min infusion	60	Hu^141^
Treatment‐resistant depression	0.5 mg/kg, 40‐min infusion	120	Singh^142^
Treatment‐resistant depression	0.5 mg/kg, 40‐min infusion	120	Phillips^4^
Treatment‐resistant depression	0.5 mg/kg, 40‐min infusion	40	Chen^143^
Treatment‐resistant depression	1 mg/kg oral ketamine	40	Domany^144^
Major depressive disorder	0.5 mg/kg, 40‐min infusion	230	Salvadore^46^

**TABLE 2:** Summary of the rapid anti‐depressant‐like effects of ketamine in animal

Species	Behavioural test		Time	Source
C57BL/6 mice	Sucrose consumption test Forced swim test Novelty‐suppressed feeding test Elevated plus maze	3 mg/kg ip	30 min	Autry^50^
C57BL/6 mice	Forced swim test Novelty‐suppressed feeding test	3 mg/kg ip	30 min	Gideons^145^
Mice	Forced swim test	2.5 mg/kg ip	30 min	Maeng^146^
C57BL/6 mice	Tail suspension test Forced swim test Sucrose preference test	10 mg/kg ip	120 min	Zhang^147^
Sprague‐Dawley rat	Forced swim test	15 mg/kg ip	120 min	Silva^148^
CD1 mice	Forced swim test	10 mg/kg ip	60 min	Clarke^102^
C57BL/6J mice	Forced swim test	10 mg/kg ip	1 d	Fitzgerald^149^
CD1 mice	Forced swim test	5 or 10 mg/kg ip	60 min	Landrigan^150^
Sprague‐Dawley rat	Forced swim test	10 mg/kg ip	30 min	Zhang^151^
Sprague‐Dawley rat	Forced swim test	10 or 30 mg/kg ip	40 min	Podkowa^152^
C57BL/6N mice	Forced swim test	10 mg/kg ip	30 min	Petryshen^153^
NMRI mice	Forced swim test Tail suspension test	3 mg/kg ip	60 min	Kordjazy^154^

## 2 NEURAL CIRCUIT

**FIGURE 1:**
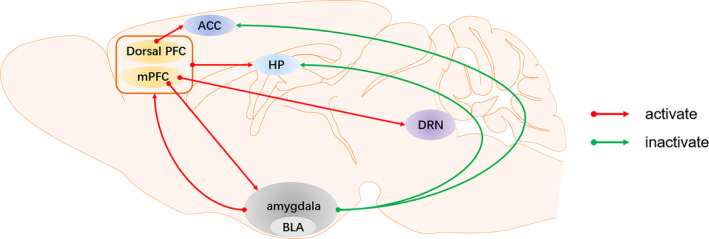
The neural circuits of depression affected by ketamine. ACC, anterior cingulate gyrus; BLA, basolateral amygdala; Dorsal PFC, dorsal pre‐frontal cortex; DRN, dorsal raphe nucleus; HP, hippocampus; mPFC, medial pre‐frontal cortex

Depression is associated with multiple brain regions including pre‐frontal cortex (PFC), hippocampus (HP) and amygdala.^30^ These regions do not play a separate role in the onset of depression but are connected by nerve fibres, forming different neural circuits. The structure and function of these circuits are abnormal under a condition of depression.^31^, ^32^, ^33^ Restoring normal connections of neural pathways may be an effective and fast way to alleviate depression symptoms. Ketamine is a non‐specific NMDAR antagonist. It can change the local activities of relevant brain regions and reshape the brain circuit in a short time (Figure 1).

### 2.1 Neural circuits associated with pre‐frontal cortex

Pre‐frontal cortex is related to cognitive function and emotional regulation.^34^ Reduced activity of PFC has been observed both in depressed patients and in rodent models of depression. Dysfunction of the pre‐frontal‐hippocampal (PFC‐HP) circuit is associated with major depression. It was demonstrated that in rat brain, functional connectivity within the PFC‐HP system is increased by acute ketamine stimulation in a dose‐ and exposure‐dependent manner.^35^ In the same way, the activation of ventral hippocampus (vHipp)‐mPFC pathway was proved to be necessary in anti‐depressant responses of ketamine.^36^

Abnormal functional connection within dorsal PFC and anterior cingulate gyrus (ACC) is highly correlated with depression.^37^ Ketamine has a positive effect on this connection. Study showed that functional connection between the right PFC and subgenual cingulate was increased in depressed patients 1 day after a single infusion of ketamine.^38^

Besides, the functional connection between the PFC and the amygdala also relates to depressive behaviour. It was reported that ketamine strengthens amygdala inputs to basal dendrites of layer V cells in mPFC and reversed depression‐like behaviours.^39^ Optogenetic experiment showed that light‐activated mPFC‐basolateral amygdala (BLA) projection produced rapid anti‐depressant‐like effects. Light stimulation to D1 dopamine receptor (Drd1) neurons in the brain region of mPFC increased the neuronal activity in the BLA area exclusively, indicating that the Drd1 neurons mediated BLA area to participate in the rapid anti‐depressant‐like effects.^40^ However, whether ketamine stimulates mPFC and amygdala in the same time has not been proved.

In addition, the PFC‐dorsal raphe nucleus (DRN) circuit has been confirmed to be implicated in depression.^41^, ^42^, ^43^ The mPFC is one of the various areas projecting densely to the DRN,^44^ which has abundant 5‐HT cell bodies located in. Activation of 5‐HT neurons can improve depression‐like behaviours in elevated plus maze and forced swim test (FST).^45^ Combining whole‐cell recordings with optogenetic approaches, it was found that the mPFC axon monosynapse was connected with 5‐HT neurons and GABAergic neurons in the DRN.^46^ The mPFC pyramidal cell, projecting to 5‐HT neurons in DRN, is a kind of glutamatergic neuron. The action potential of pyramidal cells is controlled by GABA interneurons. Ketamine blocks NMDAR located on GABA interneurons, leading to decrease in GABA activity, facilitating the firing activity of pyramidal cells and inducing glutamate release. As a result, high level of extracellular glutamate activates the post‐synaptic AMPAR.^47^, ^48^ In a word, ketamine activates 5‐HT neurons in DRN and increases the release of 5‐HT by stimulating AMPAR in mPFC.

### 2.2 Neural circuits associated with ventral tegmental area

Anhedonia, which is related to structure and function abnormalities of the reward circuit, is a core clinical feature of award‐control disorder and also a core symptom of depression. The ventral tegmental area (VTA) is a heterogeneous brain region, mainly composed of dopaminergic (DAergic) neurons (60%‐65%).^49^ VTA projects to mPFC and nucleus accumbens (NAc) and forms the mesolimbic dopamine system with the latter one. The mesolimbic dopamine system is related to depression. Studies have shown that DAergic neurons in the VTA‐NAc circuit directly participated in the regulation of coding and expressing of depressive behaviour with anhedonia.^50^, ^51^ Animal experiments demonstrated that stress could activate VTA DAergic neurons and stimulate DAergic transmission to the NAc.^52^ Similarly, clinical evidence proved that ketamine was able to increase activity in VTA, and this effect persisted for 1 week after ketamine injection, accompanied by depression‐like behaviour improved.^53^ VTA‐NAc circuit may be considered to contribute to the pathophysiology and symptomatology of depression, but whether the rapid anti‐depressant‐like effects of ketamine works through VTA‐NAc circuit is lack of evidence.

### 2.3 Neural circuits associated with lateral habenula

Lateral habenula (LHb), located in the epithalamus, is a component of the habenula nucleus. It is the main relay station for transmitting information between the marginal forebrain and midbrain. It can control the midbrain reward pathway and mediate the transmission of negative feedback information of dopamine neurons in marginal forebrain and midbrain marginal. It is also closely related to 5‐HT system. On the one hand, the indirect excitatory glutamate projection of LHb to ventral tegmental area DAergic neurons was closely related to learned helplessness behaviour in rats. In learned helplessness model, excitatory synapses projected by LHb neurons into VTA were enhanced, leading to an increased probability of pre‐synaptic release.^54^ On the contrary, stimulating GABAergic neurons would mediate inhibitory synaptic transmission, subsequently inhibiting the post‐synaptic discharge of LHb neurons and increasing the spontaneous discharge rate of VTA DAergic neurons.^55^ On the other hand, most DRN serotonergic neurons received monosynaptic glutamatergic inputted from LHb, suggesting that LHb could bidirectionally regulate the activity of 5‐HT neurons in DRN.^56^ The above two experiments applied the methods of optogenetics and chemical genetics, respectively, to identify LHb‐related neural projections function in depression. At present, there is little evidence on ketamine acting on LHb‐related circuits. Nevertheless, a recent study found that abnormal clustered excitatory post‐synaptic potentials appeared in the medial and LHb nucleus in congenitally learned helpless (cLH) rats and chronic‐restraint stress (CRS) mice. Ketamine could block the clustered discharge pattern in the LHb and improve the symptoms of depression rapidly. The mechanism was associated with NMDAR and low‐voltage‐sensitive T‐type calcium channels (T‐VSCCs). In the study, ketamine but not AMPAR antagonist NBQX eliminated the burst firing in the LHb of cLH rats and rescued the depression‐like behaviours quickly. The same results could be seen in specific NMDAR antagonist 2‐amino‐5‐phosphonopentanoic acid (AP5) and T‐VSCCs blocker mibefradil and ZD7288. Moreover, bilateral infusion of mibefradil into the LHb of cLH rats and systematic injection of the T‐VSCCs blocker 2‐ethyl‐2‐methylsuccinimide (ethosuximide) in CRS mice elicited rapid anti‐depressant effects.^57^ According to this research, blocking T‐VSCCs may produce rapid anti‐depressant effects. Nevertheless, ethosuximide did not exert the same potent anti‐depressant effects in CSDS‐susceptible mice^58^ or non‐medicated adult MDD patients.^59^ Differences exist in different depressive animal models since the pathogenesis is diverse. More than that, the internal environment of the human body is more complicated than that of animal. Even one pathway is affected by ketamine, other alternatives can be activated instead. Studies on other T‐VSCCs blockers and the possible targets need to be done.

### 2.4 Neural circuits associated with amygdala

The amygdala is involved in coordinating the function of cortical networks when evaluating the biological significance of affective stimuli. Liu et al^39^ discovered that ketamine activated amygdala and increased the amygdala output to the PFC through the anterior marginal area in the chronic unpredictable stress (CUS) model of rats. By using fMRI and resting‐state fMRI (rsfMRI), it was found that in healthy subjects without any mental, neurological or medical illness, ketamine reduced neural reactivity in the bilateral amygdalo‐hippocampal complex during emotional stimulation, which was different from amygdala‐PFC circuit.^60^

It is hypothesized that the amygdala and its interaction with the pre‐genual anterior cingulate cortex (pgACC) could predict the response of patients to ketamine. Clinical studies have demonstrated that MDD patients were either in working memory task mode or stimulated by rapidly presenting fearful faces, and the pgACC was highly activated but could be inactivated by ketamine within 4 hours. Pre‐treated with ketamine, patients with the lowest pgACC activation had the greatest improvement in depressive symptoms when working memory load increased. Moreover, the functional connection between the pgACC and the amygdala was negatively correlated with the change in anti‐depressant symptoms.^61^, ^62^ Notably, another study showed that a single bilateral infusion of (R)‐ketamine into basolateral amygdala and central nucleus of the amygdala had no anti‐depressant effects.^63^ Unlike (R)‐ketamine, (S)‐ketamine induced acute proteomic changes in the amygdala in wild mice after 2 hours, which may contribute to its the fast antidepressant effects.^64^ In clinical trial, it was found that (S)‐ketamine decreased then the connectivity among the amygdala, ACC and insula.^65^ Maybe (S)‐ketamine is the key to the function of (R,S)‐ketamine on the amygdala.

## 3 SYNAPTIC PLASTICITY

Another crucial mechanism of ketamine rapid anti‐depressant‐like effects is synaptic plasticity. Synapse is the basic structure of information transmission and processing between neurons. Synaptic plasticity, including changes in the number, structure and function of synapse, is a kind of adaptive change which enables brain to do self‐repair. It is critically important for individuals to maintain normal functions when facing changing internal and external environments. Synaptic plasticity includes long‐term potentiation (LTP) and long‐term depression (LTD). Stress can interfere with the normal balance in synaptic plasticity, inhibiting LTP and/or promoting LTD, resulting in synaptic weakening and neuronal atrophy. Impairment of synaptic plasticity in hippocampus and pre‐frontal cortex is particularly pronounced in depression.^66^

### 3.1 Classical mechanisms of synaptic plasticity

**FIGURE 2:**
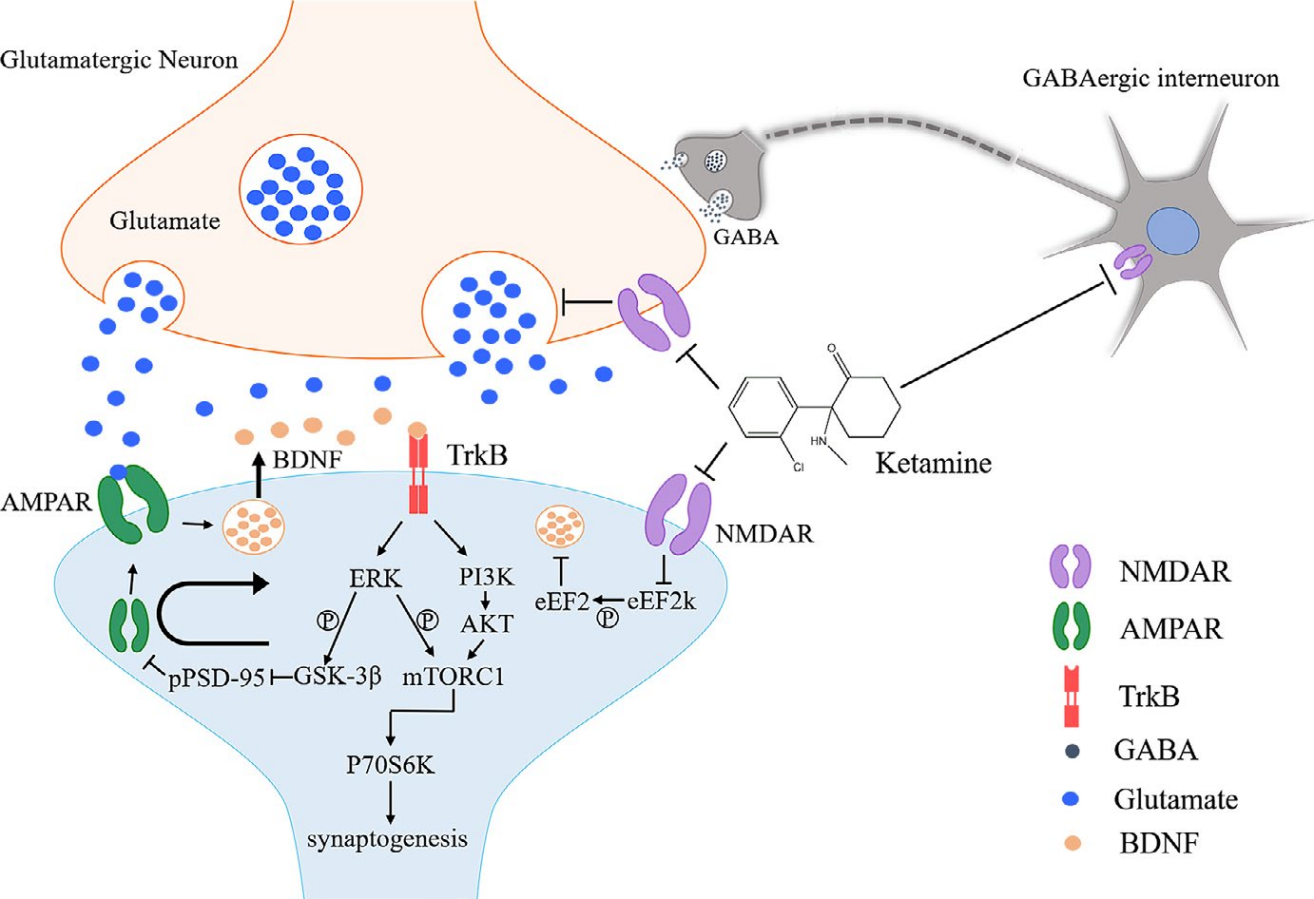
Proposed mechanisms of ketamine act on synaptic plasticity. AMPAR, α‐amino‐3‐hydroxy‐5‐methyl‐4‐isoxazole‐propionic acid receptor; BDNF, brain‐derived neurotrophic factor; eEF2, eukaryotic elongation factor 2; GSK‐3β, glycogen synthase kinase‐3β; mTORC1, mechanistic target of rapamycin complex 1; NMDAR, *N*‐methyl‐d‐aspartate receptor; P70S6K, P70S6 kinase; PSD‐95, post‐synaptic density‐95; TrkB, tropomyosin receptor kinase B

*N*‐methyl‐d‐aspartate receptor is ionotropic glutamate receptors and widely distributed in the central nervous system. It is a heterotetramer with subunits including GluN1, GluN2A, GluN2B, GluN2C, GluN2D, GluN3A and GluN3B.^67^ NMDAR is ion channels of Na^+^ and Ca^2+^. Under physiological conditions, the permeability of NMDAR is blocked by Mg^2+^ in resting state. When stimulated, glutamate released by the pre‐synaptic membrane acts on AMPAR and enhances its ion flow, releasing Mg^2+^ and unblocking the NMDA receptor channel. Then, a large amount of Ca^2+^ goes into neurons, resulting in excitatory toxicity and death of nerve cells. Ketamine acts on NMDAR and blocks the influx of Ca^2+^, resulting in neurons survival and reversion of synaptic structural defect. Activation or inhibition of NMDAR triggers a series of cascades, altering expression level and function of AMPAR, leading to decrease or increase in AMPAR‐mediated synaptic transmission BDNF. Meanwhile, inhibition of NMDAR also leads to inactivation of eukaryotic elongation factor 2 (eEF2), resulting in reducing eEF2 phosphorylation and enhancing BDNF protein synthesis^68^ and regulating synaptogenesis (Figure 2). Other drugs also produce anti‐depressant effects. Cannabidiol, neuropeptide VGF (non‐acronymic) C‐terminal peptide TLQP‐62 and NV‐5138 increased activity of BDNF‐mTOR signalling in the mPFC to induce rapid anti‐depressant effects.^69^, ^70^, ^71^ d‐Methadone is a non‐competitive NMDAR antagonist and could decrease immobility of rats in FST in 24 hours.^72^ Another NMDAR blocker Ro 25‐6981 also exhibited anti‐depressant effects in pre‐clinical and clinical tests.^73^, ^74^ However, a meta‐analysis showed that non‐ketamine NMDAR antagonists were superior to placebo only on days 5‐8, while ketamine reduced depression in 40 minutes.^75^ Not all non‐ketamine NMDAR antagonists elicit robust anti‐depressant effects such as ketamine, suggesting that NMDAR may not be the key role in the anti‐depressant mechanisms of ketamine. Inhibition of NMDAR causes changes in its downstream molecules and signalling pathways, and these changes can be seen in depression‐related brain regions.^76^ But now, there are more and more reports of rapid anti‐depressants that are less related to NMDAR. Maybe we should stop focusing on NMDAR only and begin to pay more attention to other potential targets. Other mechanisms of anti‐depressant effects of ketamine will be discussed below.

#### 3.1.1 AMPAR in synaptic plasticity

AMPAR belongs to the ionic glutamate receptor and is dynamically expressed in the post‐synaptic membrane. It mediates rapid excitatory synaptic transmission in the central nervous system and is related to induction and maintenance of LTP and LTD.^77^, ^78^, ^79^, ^80^ Increasing insertion and phosphorylation of AMPAR leads to LTP and increases the sensitivity of glutamate to synaptic transmission.^81^ NMDAR antagonists facilitate glutamate release and increase synaptic glutamate concentration by blocking NMDARs on pre‐synaptic neurons or GABA interneurons. On the one hand, inhibiting the pre‐synaptic NMDARs leads to a release of glutamate from pre‐synaptic neurons. On the other hand, suppressing the NMDARs on GABA interneurons will decrease the activity of GABA interneurons and disinhibit the pre‐synaptic neurons.^82^, ^83^, ^84^ Glutamate can activate AMPAR and downstream signalling pathways. On the one hand, BDNF in post‐synaptic neurons will be released into the synaptic cleft immediately after AMPARs are activated, activating the TrkB on the post‐synaptic membrane.^85^ Then, the activation of TrkB increases the phosphorylation level of glycogen synthase kinase 3‐β (GSK‐3β) via ERK signalling pathway, leading to a decrease in the phosphorylation level of post‐synaptic density‐95 (PSD‐95) and the internalization of the AMPA GluA1 subunit, allowing ketamine to enhance signalling through the AMPAR^86^ and promote synapse generation.^87^ On the other hand, the downstream ERK and PI3K‐AKT signalling pathways activate and stimulate mechanistic target of rapamycin complex 1 (mTORC1) phosphorylation to promote synapse formation.^88^ Subsequently, the phosphorylation level of P70S6 kinase (P70S6K) increases, resulting in synaptogenesis.^89^ These results induced by ketamine could be eliminated by AMPAR antagonists and mimicked by AMPA‐positive allosteric modulator CX614.^88^

#### 3.1.2 BDNF in synaptic plasticity

Brain‐derived neurotrophic factor is a vital protein in the process of synaptic transmission. It regulates neural plasticity, synaptic production, neurogenesis and cell survival. BDNF is necessary for the formation and maintenance of activity‐dependent synaptic connections. It has been found that the expression of BDNF in the pre‐frontal cortex and hippocampus was downregulated in animal depression models, so as the level of BDNF in depressed patients.^90^, ^91^ Evidence showed that ketamine administration increases BDNF levels and improves depressive‐like behaviours.^92^, ^93^, ^94^ More importantly, BDNF is indispensable in anti‐depressant effects. In the BDNF Met gene knock‐in mice, especially Met/Met mice, synaptogenesis was significantly weakened,^95^ consisted of depressed patients.^96^ Clinical study showed that either 0.5 or 0.2 mg/kg of ketamine injection could reduce suicidal ideation of patients who had the Val allelic genes. However, patients with genotype Met/Met only responded at a dose of 0.5 mg/kg ketamine.^96^ Sufficient BDNF content regulates synaptic plasticity and participates in reversing depression.^97^, ^98^, ^99^

**FIGURE 3:**
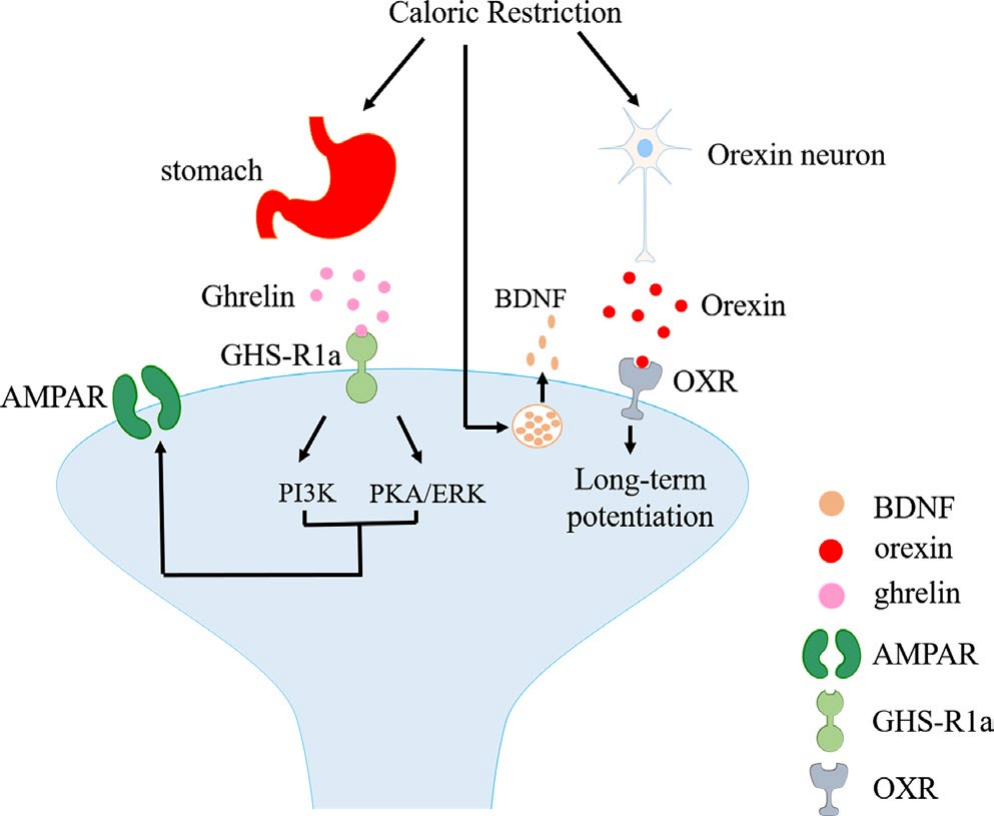
Proposed mechanisms of CR act on synaptic plasticity. AMPAR, α‐amino‐3‐hydroxy‐5‐methyl‐4‐isoxazole‐propionic acid receptor; BDNF, brain‐derived neurotrophic factor; ERK, extracellular signal‐regulated kinase; GHS‐R1a, growth hormone secretagogue receptor 1a; PI3K, phosphatidylinositol 3‐kinase; PKA, protein kinase A

Except for ketamine, acute caloric restriction (CR) is also able to elevate BDNF level. CR refers to a 30%‐40% reduction in calorie intake while retaining protein, vitamins, minerals and water intake to maintain proper nutrition. Some mental illnesses, such as the typical major depression and anorexia nervosa, are characterized by reduced calorie intake. Previous studies showed that long‐term strict energy limitation (5 weeks, 50% intake of the control group) may cause brain 5‐HT system dysfunction, leading to the development of depression and anxiety.^100^ Otherwise, strict energy limitation might lead to malnutrition^101^ and other metabolic dysfunctions in the body. Our group found that 9‐hour acute CR increased BDNF level in the PFC and hippocampus, resulting in neurogenesis in the subgranular region and producing anti‐depressant‐like effects.^27^ Aiming to figure out whether the anti‐depressant effects of CR are related to the 5‐HT system, we combined CR with imipramine and 5‐HT_2A/2C_ receptor agonist (±)‐1‐(2,5‐dimethoxy‐4‐iodophenyl)‐2‐aminopropane hydrochloride (DOI) for authentication. The results showed that DOI could partially reverse the anti‐depressant effects of imipramine and 9‐hour CR.^28^ We also found that DOI could suppress the increase in BDNF level and 5‐HT_2A_R antagonist ketanserin inhibited the effects of DOI on BDNF.^102^ There is a possibility that acute fasting may exert anti‐depressant effects by blocking 5‐HT_2A_R. Evidence shows that the activation of 5‐HTergic system leads to an activation of glutamatergic system. Activated by 5‐HT receptors, glutamate pyramidal cells in mPFC release BDNF rapidly and activate BDNF signalling pathway, resulting in synaptogenesis accompanied by rapid anti‐depressant effects.^103^, ^104^, ^105^ These studies suggest that monoamine manner (5‐HT) and non‐monoamine manner (BDNF) are not separated in anti‐depressant effects. This suggests us that combining monoamine with non‐monoamine may be a new strategy for treating MDD. Some studies showed that CR regulated the release of orexin^106^, ^107^, ^108^, ^109^ and ghrelin,^110^, ^111^, ^112^, ^113^, ^114^, ^115^, ^116^, ^117^ producing some anti‐depressant effects. But this evidence on synaptic plasticity is weak, and we mention here only for reference (Figure 3).

Additionally, scopolamine has similar pharmacological mechanisms to ketamine for its anti‐depressant effects. Scopolamine activates AMPARs, promotes BDNF release rapidly and stimulates BDNF‐mTOR signalling pathway.^118^ The difference is that scopolamine acts on cholinergic system. Scopolamine inhibits GABAergic neuron function by combining with M1‐AChR on GABA interneurons in mPFC.^119^

### 3.2 Neuroglia in synaptic plasticity

Ketamine also affects glial cells in the central nervous system to regulate synaptic plasticity. Glial cells are mainly divided into three categories: astroglia, microglia and oligodendroglia. Among them, the former two are associated with depression. Astroglia is the most abundant glial cell. Its main functions are to regulate regional blood flow and energy metabolism, immune defence and amino acid neurotransmitter clearance. It is also associated with the stabilization and dissection of synaptic connections^120^ and participates in anti‐depressant effects.^121^ Pre‐treated with ketamine 1 day after, immobility time in FST was significantly reduced. The volume of CA1 stratum radiatum and molecular layer of the dentate gyrus in the hippocampus and the volume of astrocytes of rats increased significantly, so as the number and length.^122^ Ketamine modified the morphology of astrocytes and astrocytes, regulating the synaptic microenvironment, neurogenesis and angiogenesis.^123^ Microglia is a kind of immunocompetent cell. Excessive microglial activation would cause inflammatory process, leading to astrocyte glutamatergic dysfunction and activation of microglial function in turn.^124^ Evidence showed that ketamine inactivates microglial due to inhibition of ERK1/2 phosphorylation.^125^ Besides, ketamine regulated STAT3 and the type I interferon pathway in microglia through eEF2, increasing the BDNF expression and promoting the synthesis of PSD95 and synapsin I (SYN1).^126^ Additionally, microglial cells induce immune dysfunction by producing quinolinic acid (QUIN). QUIN is an endogenous modulator with agonistic properties on NMDA. It was observed that in acutely depressed patients, QUIN increased in subregions of the anterior cingulate gyrus.^127^ Increase in QUIN comes along with decrease in kynurenic acid (KYNA), a NMDA receptor antagonist synthesized by astrocytes.^128^ Ketamine could modulate the microglial reactivity and decrease QUIN production. It was reported that KYNA‐to‐QUIN ratio was a predictor of ketamine response in treatment‐resistant depressed patients, while the reduction in QUIN after treated by ketamine was a predictor to the reduction in MADRS score.^129^ Ketamine regulates functions of astrocytes and microcytes to maintain synaptic complement.

### 3.3 Neuroinflammation in synaptic plasticity

Depression is considered to be relevant with the activation of chronic, low‐grade inflammatory responses and cell‐mediated immunity.^130^, ^131^ Chronic inflammatory reactions cause neurons apoptosis in brain regions associated with emotion regulation such as hippocampus,^132^, ^133^ leading to impairment of synaptic plasticity. Ketamine could normalize abnormal neurobehaviours induced by neuroinflammation through regulating the interleukin (IL)‐1β, tumour necrosis factor (TNF)‐α and IL‐6.^134^ In rodent model, ketamine would reverse the increase in IL‐1β and TNF‐α caused by lipopolysaccharide (LPS), shortening the immobility time significantly in FST and promoting hippocampal neurogenesis.^135^ In addition, ketamine also plays an anti‐depressant part in the central nervous system by regulating the immune system's immune response. It promoted the conversion of macrophages in CNS into M2‐type cells with anti‐inflammatory properties, reversing the inflammatory response through NMDAR and mTOR.^136^ Zhang et al found that the desperate behaviours of susceptible mice in the social defeat stress model were improved in FST and tail suspension test (TST) after receiving intravenous injection of the inflammatory factor IL‐6 receptor antibody MR16‐1. MR16‐1 treatment increased the expression of PSD95 and AMPAR1, so as the dendritic spines in hippocampus, and PFC and NAc in susceptible mice. Besides, MR‐16 normalized the components of gut microbiota in susceptible mice by downregulating the level of IL‐6 in the periphery.^137^ Changes in peripheral IL‐6 and gut microbiota may be vital for the pathogenesis of depression. It was found that baseline serum levels of IL‐6 were both higher in ketamine responder and non‐responder groups than control group. More than that, serum level of IL‐6 is significantly higher in the responder group than non‐responder group.^138^ Another clinical study also demonstrated that higher baseline interleukin‐6 (IL‐6) in serum predicted better response to ketamine.^139^ Serum IL‐6 may be a predictive biomarker for the anti‐depressant effects of ketamine in TRD patients.

### 3.4 A1R in synaptic plasticity

**FIGURE 4:**
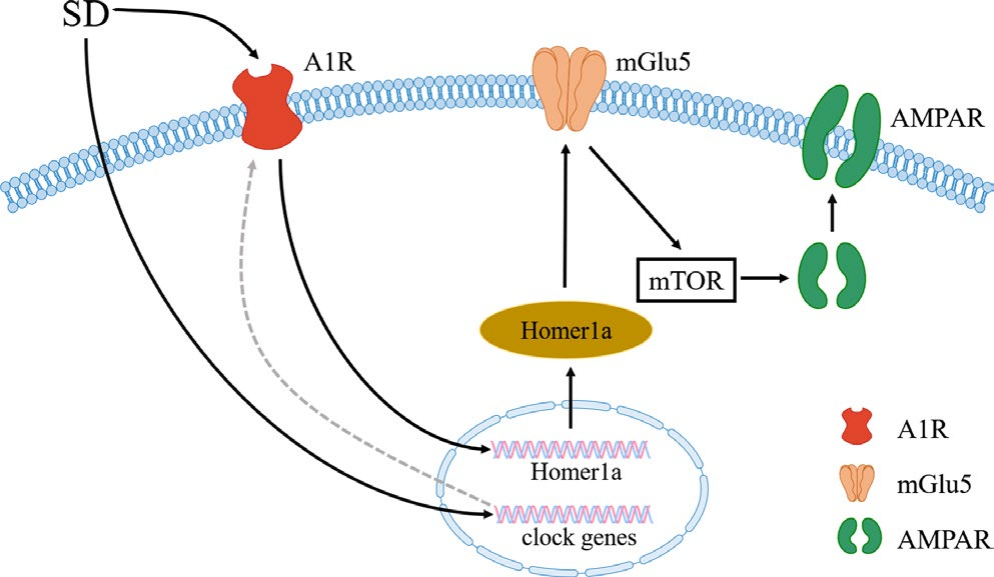
Proposed mechanisms of SD act on synaptic plasticity. A1R, A1 receptors; AMPAR, α‐amino‐3‐hydroxy‐5‐methyl‐4‐isoxazole‐propionic acid receptor; mGlu5, metabotropic glutamate receptor 5; mTOR, mechanistic target of rapamycin; SD, sleep deprivation

A1 receptors (A1R) are of high affinity with adenosine and are distributed both pre‐ and post‐synaptically. A1R is essential for sleep deprivation (SD) to exert rapid anti‐depression‐like effects. Therapeutic SD is a direct and rapid treatment for MDD, reducing the depressive symptoms of 50%‐60% of MDD patients significantly within a few hours,^140^ consistent with animal experiment.^141^ It was reported that SD produced rapid anti‐depressant effects by activating adenosine A1R in astrocytes and could be mimicked by the application of A1 agonist CCPA.^142^ A1R exerts anti‐depressant‐like effects by regulating synaptic plasticity through Homer1a. Homer1a is a kind of synaptic protein upregulated by ketamine and SD, and the upregulation of Homer1a produces rapid anti‐depressant‐like effects. When Homer1a was knocked out in mPFC, the upregulation of A1R and the anti‐depressant effects of SD were inhibited.^143^, ^144^ TAT‐Homer1a, which is a fusion of the HIV TAT peptide with full‐length Homer1a protein, has brain and membrane permeability. The application of TAT‐H1A in vivo and in vitro increased the level of Homer1a and enhanced metabotropic glutamate receptor 5 (mGlu5) signal transduction. As a result, phosphorylation of the mTOR increased and the expression and activity of AMPAR were elevated.^145^ The molecular change was consistent with those caused by ketamine and also SD. In animal studies, AMPAR level in the cerebral cortex and hippocampus was about 40% higher after arousal than after sleep. The change in AMPAR phosphorylation and other enzymes important for plasticity was consistent with synaptic strengthening during wakefulness and contraction during sleep.^146^ These evidence indicates that synaptic homeostasis is regulated by wakefulness and sleep. Synaptic homeostasis refers to the ability of neurons to regulate their own excitability and synaptic strength, connected closely with synaptic plasticity. The core of the synaptic homeostasis hypothesis is that the number and intensity of cortical synapses vary widely throughout the sleep‐wake cycle. It is believed that wakefulness leads to a net increase in synaptic strength of the cortical circuits, while a basic function of sleep is to reduce the proportion of cortical synapses.^147^ Given to that, circadian rhythms also regulate synaptic plasticity. Circadian rhythms are reset by the transcription of clock genes, including the cycle genes PER1, PER2 and PER3. After 2‐hour SD treatment on mice, the expression levels of PER1 and PER2 significantly increased.^148^ Similarly, ketamine regulated circadian rhythms by affecting clock genes accompanied by a rapid anti‐depressant effect. In animal experiment, it was seen that clock genes including PER2, neuronal PAS domain protein 4 and D‐Box binding protein, were downregulated in mice treated with ketamine and SD.^149^ Reviewing data from human, animal and neuronal cell, both low‐dose SD and ketamine could regulate circadian rhythms.^150^ It is hypothesized that A1R ameliorates the depression‐like behaviours through regulating cycle genes and then affecting synaptic homeostasis.^151^ However, we still lack evidence for that so far (Figure 4).

## 4 CONCLUSION

In this review, we summarized the mechanisms of rapid anti‐depressant‐like effects induced by ketamine, CR and SD. Rapid anti‐depressant‐like effect is a result of mutual regulation of neural circuits and synaptic plasticity. On the one hand, rebuilding the neurotransmitter balance by regulating the levels of dopamine and serotonin can reshape neural circuits. On the other hand, glial cells, hormones and related receptors regulate the microenvironment and synaptic homeostasis. As a result, the functions and connections of various areas in the brain that regulate emotion return to normal. Clinically, the symptoms of depression are alleviated. Rapid anti‐depressant drugs and behavioural interventions bring a glimmer of hope to it. Although depression is a refractory disease and there exist many unknowns in the pathogenesis of depression, with the application of optogenetics and the discovery of crosstalk in different pathways, more and more safe and effective rapid anti‐depressant treatments are about to occur.

## CONFLICT OF INTEREST

The authors declare no conflict of interest.

## AUTHOR CONTRIBUTIONS

FP wrote the first draft. JF, TG and QL participated in the discussion of the manuscript. BL provided critical revisions. All authors approved the final version of the manuscript for submission.

## Data Availability

Data available on request.

## References

[1] Kessler RC , Bromet EJ . The epidemiology of depression across cultures. Annu Rev Public Health. 2013;34:119‐138. 23514317 10.1146/annurev-publhealth-031912-114409 PMC4100461

[2] Malhi GS , Mann JJ . Depression . Lancet. 2018;392(10161):2299‐2312. 30396512 10.1016/S0140-6736(18)31948-2

[3] Mrazek DA , Hornberger JC , Altar CA , Degtiar I . A review of the clinical, economic, and societal burden of treatment‐resistant depression: 1996–2013. Psychiatr Serv. 2014;65(8):977‐987. 24789696 10.1176/appi.ps.201300059

[4] Phillips JL , Norris S , Talbot J et al. Single and repeated ketamine infusions for reduction of suicidal ideation in treatment‐resistant depression. Neuropsychopharmacology. 2020;45(4):606‐612. 31759333 10.1038/s41386-019-0570-x PMC7021716

[5] Berman RM , Cappiello A , Anand A et al. Antidepressant effects of ketamine in depressed patients. Biol Psychiatry. 2000;47(4):351‐354. 10686270 10.1016/s0006-3223(99)00230-9

[6] Insel TR , Scolnick EM . Cure therapeutics and strategic prevention: raising the bar for mental health research. Mol Psychiatry. 2006;11(1):11‐17. 16355250 10.1038/sj.mp.4001777 PMC1586099

[7] Aan Het Rot M , Zarate CA Jr , Charney DS , Mathew SJ . Ketamine for depression: where do we go from here? Biol Psychiatry. 2012;72(7):537‐547. 22705040 10.1016/j.biopsych.2012.05.003 PMC3438349

[8] Ebert B , Mikkelsen S , Thorkildsen C , Borgbjerg FM . Norketamine, the main metabolite of ketamine, is a non‐competitive NMDA receptor antagonist in the rat cortex and spinal cord. Eur J Pharmacol. 1997;333(1):99‐104. 9311667 10.1016/s0014-2999(97)01116-3

[9] Canuso CM , Singh JB , Fedgchin M et al. Efficacy and safety of intranasal esketamine for the rapid reduction of symptoms of depression and suicidality in patients at imminent risk for suicide: results of a double‐blind, randomized placebo‐controlled study. Am J Psychiatry. 2018;175(7):620‐630. 29656663 10.1176/appi.ajp.2018.17060720

[10] Popova V , Daly EJ , Trivedi M et al. Efficacy and safety of flexibly dosed esketamine nasal spray combined with a newly initiated oral antidepressant in treatment‐resistant depression: a randomized double‐blind active‐controlled study. Am J Psychiatry. 2019;176(6):428‐438. 31109201 10.1176/appi.ajp.2019.19020172

[11] Daly EJ , Singh JB , Fedgchin M et al. Efficacy and safety of intranasal esketamine adjunctive to oral antidepressant therapy in treatment‐resistant depression: a randomized clinical trial. JAMA Psychiatry. 2018;75(2):139‐148. 29282469 10.1001/jamapsychiatry.2017.3739 PMC5838571

[12] Turner EH . Esketamine for treatment‐resistant depression: seven concerns about efficacy and FDA approval. Lancet Psychiatry. 2019;6(12):977‐979. 31680014 10.1016/S2215-0366(19)30394-3

[13] Hashimoto K . Rapid‐acting antidepressant ketamine, its metabolites and other candidates: a historical overview and future perspective. Psychiatry Clin Neurosci. 2019;73(10):613‐627. 31215725 10.1111/pcn.12902 PMC6851782

[14] Zhang JC , Li SX , Hashimoto K . R (‐)‐ketamine shows greater potency and longer lasting antidepressant effects than S (+)‐ketamine. Pharmacol Biochem Behav. 2014;116:137‐141. 24316345 10.1016/j.pbb.2013.11.033

[15] Yang C , Shirayama Y , Zhang J‐C et al. R‐ketamine: a rapid‐onset and sustained antidepressant without psychotomimetic side effects. Transl Psychiatry. 2015;5:e632. 26327690 10.1038/tp.2015.136 PMC5068814

[16] Fukumoto K , Toki H , Iijima M et al. Antidepressant potential of (R)‐ketamine in rodent models: comparison with (S)‐ketamine. J Pharmacol Exp Ther. 2017;361(1):9‐16. 28115553 10.1124/jpet.116.239228

[17] Masaki Y , Kashiwagi Y , Watabe H , Abe K . (R)‐ and (S)‐ketamine induce differential fMRI responses in conscious rats. Synapse. 2019;73(12):e22126. 31397936 10.1002/syn.22126

[18] Vollenweider FX , Leenders KL , Oye I , Hell D , Angst J . Differential psychopathology and patterns of cerebral glucose utilisation produced by (S)‐ and (R)‐ketamine in healthy volunteers using positron emission tomography (PET). Eur Neuropsychopharmacol. 1997;7(1):25‐38. 9088882 10.1016/s0924-977x(96)00042-9

[19] Yang C , Yang J , Luo A , Hashimoto K . Molecular and cellular mechanisms underlying the antidepressant effects of ketamine enantiomers and its metabolites. Transl Psychiatry. 2019;9(1):280. 31699965 10.1038/s41398-019-0624-1 PMC6838457

[20] Yang C , Ren Q , Qu Y et al. Mechanistic target of rapamycin‐independent antidepressant effects of (R)‐ketamine in a social defeat stress model. Biol Psychiatry. 2018;83(1):18‐28. 28651788 10.1016/j.biopsych.2017.05.016

[21] Chang L , Zhang K , Pu Y et al. Comparison of antidepressant and side effects in mice after intranasal administration of (R, S)‐ketamine, (R)‐ketamine, and (S)‐ketamine. Pharmacol Biochem Behav. 2019;181:53‐59. 31034852 10.1016/j.pbb.2019.04.008

[22] Neasta J , Barak S , Hamida SB , Ron D . mTOR complex 1: a key player in neuroadaptations induced by drugs of abuse. J Neurochem. 2014;130(2):172‐184. 24666346 10.1111/jnc.12725 PMC4107045

[23] Hashimoto K , Kakiuchi T , Ohba H , Nishiyama S , Tsukada H . Reduction of dopamine D2/3 receptor binding in the striatum after a single administration of esketamine, but not R‐ketamine: a PET study in conscious monkeys. Eur Arch Psychiatry Clin Neurosci. 2017;267(2):173‐176. 27091456 10.1007/s00406-016-0692-7 PMC5323469

[24] Ren Z , Yan P , Zhu L et al. Dihydromyricetin exerts a rapid antidepressant‐like effect in association with enhancement of BDNF expression and inhibition of neuroinflammation. Psychopharmacology. 2018;235(1):233‐244. 29058041 10.1007/s00213-017-4761-z

[25] Chiechio S , Canonico PL , Grilli M . l‐Acetylcarnitine: a mechanistically distinctive and potentially rapid‐acting antidepressant drug. Int J Mol Sci. 2018;19(1). 10.3390/ijms19010011 PMC5795963 29267192

[26] Fogaça MV , Fukumoto K , Franklin T et al. N‐methyl‐D‐aspartate receptor antagonist d‐methadone produces rapid, mTORC1‐dependent antidepressant effects. Neuropsychopharmacology. 2019;44(13):2230‐2238. 31454827 10.1038/s41386-019-0501-x PMC6898593

[27] Wang P , Li B , Fan J et al. Additive antidepressant‐like effects of fasting with beta‐estradiol in mice. J Cell Mol Med. 2019;23(8):5508‐5517. 31211521 10.1111/jcmm.14434 PMC6653417

[28] Li B , Zhao J , Lv J et al. Additive antidepressant‐like effects of fasting with imipramine via modulation of 5‐HT2 receptors in the mice. Prog Neuropsychopharmacol Biol Psychiatry. 2014;48:199‐206. 24036107 10.1016/j.pnpbp.2013.08.015

[29] Berger M , Vollmann J , Hohagen F et al. Sleep deprivation combined with consecutive sleep phase advance as a fast‐acting therapy in depression: an open pilot trial in medicated and unmedicated patients. Am J Psychiatry. 1997;154(6):870‐872. 9167521 10.1176/ajp.154.6.870

[30] Leuner B , Shors TJ . Stress, anxiety, and dendritic spines: what are the connections? Neuroscience. 2013;251:108‐119. 22522470 10.1016/j.neuroscience.2012.04.021

[31] Vasic N , Walter H , Sambataro F , Wolf RC . Aberrant functional connectivity of dorsolateral prefrontal and cingulate networks in patients with major depression during working memory processing. Psychol Med. 2009;39(6):977‐987. 18845009 10.1017/S0033291708004443

[32] Liao YI , Huang X , Wu Q et al. Is depression a disconnection syndrome? Meta‐analysis of diffusion tensor imaging studies in patients with MDD. J Psychiatry Neurosci. 2013;38(1):49‐56. 22691300 10.1503/jpn.110180 PMC3529219

[33] Shen X , Reus LM , Cox SR et al. Subcortical volume and white matter integrity abnormalities in major depressive disorder: findings from UK Biobank imaging data. Sci Rep. 2017;7(1):5547. 28717197 10.1038/s41598-017-05507-6 PMC5514104

[34] McEwen BS , Morrison JH . The brain on stress: vulnerability and plasticity of the prefrontal cortex over the life course. Neuron. 2013;79(1):16‐29. 23849196 10.1016/j.neuron.2013.06.028 PMC3753223

[35] Gass N , Schwarz AJ , Sartorius A et al. Sub‐anesthetic ketamine modulates intrinsic BOLD connectivity within the hippocampal‐prefrontal circuit in the rat. Neuropsychopharmacology. 2014;39(4):895‐906. 24136293 10.1038/npp.2013.290 PMC3924524

[36] Carreno FR , Donegan JJ , Boley AM et al. Activation of a ventral hippocampus‐medial prefrontal cortex pathway is both necessary and sufficient for an antidepressant response to ketamine. Mol Psychiatry. 2016;21(9):1298‐1308. 26619811 10.1038/mp.2015.176

[37] Rantamaki T , Yalcin I . Antidepressant drug action – from rapid changes on network function to network rewiring. Prog Neuropsychopharmacol Biol Psychiatry. 2016;64:285‐292. 26066070 10.1016/j.pnpbp.2015.06.001

[38] Gärtner M , Aust S , Bajbouj M et al. Functional connectivity between prefrontal cortex and subgenual cingulate predicts antidepressant effects of ketamine. Eur Neuropsychopharmacol. 2019;29(4):501‐508. 30819549 10.1016/j.euroneuro.2019.02.008

[39] Liu RJ , Ota KT , Dutheil S , Duman RS , Aghajanian GK . Ketamine strengthens CRF‐activated amygdala inputs to basal dendrites in mPFC layer V pyramidal cells in the prelimbic but not infralimbic subregion, a key suppressor of stress responses. Neuropsychopharmacology. 2015;40(9):2066‐2075. 25759300 10.1038/npp.2015.70 PMC4613616

[40] Hare BD , Shinohara R , Liu RJ , Pothula S , DiLeone RJ , Duman RS . Optogenetic stimulation of medial prefrontal cortex Drd1 neurons produces rapid and long‐lasting antidepressant effects. Nat Commun. 2019;10(1):223. 30644390 10.1038/s41467-018-08168-9 PMC6333924

[41] Warden MR , Selimbeyoglu A , Mirzabekov JJ et al. A prefrontal cortex‐brainstem neuronal projection that controls response to behavioural challenge. Nature. 2012;492:428‐432. 23160494 10.1038/nature11617 PMC5929119

[42] Pham TH , Mendez‐David I , Defaix C et al. Ketamine treatment involves medial prefrontal cortex serotonin to induce a rapid antidepressant‐like activity in BALB/cJ mice. Neuropharmacology. 2017;112(Pt A):198‐209. 27211253 10.1016/j.neuropharm.2016.05.010

[43] Fukumoto K , Iijima M , Chaki S . The antidepressant effects of an mGlu2/3 receptor antagonist and ketamine require AMPA receptor stimulation in the mPFC and subsequent activation of the 5‐HT neurons in the DRN. Neuropsychopharmacology. 2016;41(4):1046‐1056. 26245499 10.1038/npp.2015.233 PMC4748429

[44] Pollak Dorocic I , Fürth D , Xuan Y et al. A whole‐brain atlas of inputs to serotonergic neurons of the dorsal and median raphe nuclei. Neuron. 2014;83(3):663‐678. 25102561 10.1016/j.neuron.2014.07.002

[45] Teissier A , Chemiakine A , Inbar B et al. Activity of raphe serotonergic neurons controls emotional behaviors. Cell Rep. 2015;13(9):1965‐1976. 26655908 10.1016/j.celrep.2015.10.061 PMC4756479

[46] Geddes SD , Assadzada S , Lemelin D et al. Target‐specific modulation of the descending prefrontal cortex inputs to the dorsal raphe nucleus by cannabinoids. Proc Natl Acad Sci USA. 2016;113(19):5429‐5434. 27114535 10.1073/pnas.1522754113 PMC4868450

[47] Li N , Lee B , Liu R‐J et al. mTOR‐dependent synapse formation underlies the rapid antidepressant effects of NMDA antagonists. Science. 2010;329(5994):959‐964. 20724638 10.1126/science.1190287 PMC3116441

[48] Miller OH , Moran JT , Hall BJ . Two cellular hypotheses explaining the initiation of ketamine's antidepressant actions: direct inhibition and disinhibition. Neuropharmacology. 2016;100:17‐26. 26211972 10.1016/j.neuropharm.2015.07.028

[49] Nair‐Roberts RG , Chatelain‐Badie SD , Benson E , White‐Cooper H , Bolam JP , Ungless MA . Stereological estimates of dopaminergic, GABAergic and glutamatergic neurons in the ventral tegmental area, substantia nigra and retrorubral field in the rat. Neuroscience. 2008;152(4):1024‐1031. 18355970 10.1016/j.neuroscience.2008.01.046 PMC2575227

[50] Tye KM , Mirzabekov JJ , Warden MR et al. Dopamine neurons modulate neural encoding and expression of depression‐related behaviour. Nature. 2013;493(7433):537‐541. 23235822 10.1038/nature11740 PMC4160519

[51] Chaudhury D , Walsh JJ , Friedman AK et al. Rapid regulation of depression‐related behaviours by control of midbrain dopamine neurons. Nature. 2013;493(7433):532‐536. 23235832 10.1038/nature11713 PMC3554860

[52] Rada P , Moreno SA , Tucci S et al. Glutamate release in the nucleus accumbens is involved in behavioral depression during the PORSOLT swim test. Neuroscience. 2003;119(2):557‐565. 12770568 10.1016/s0306-4522(03)00162-3

[53] Sterpenich V , Vidal S , Hofmeister J et al. Increased reactivity of the mesolimbic reward system after ketamine injection in patients with treatment‐resistant major depressive disorder. Anesthesiology. 2019;130(6):923‐935. 31021848 10.1097/ALN.0000000000002667

[54] Li BO , Piriz J , Mirrione M et al. Synaptic potentiation onto habenula neurons in the learned helplessness model of depression. Nature. 2011;470(7335):535‐539. 21350486 10.1038/nature09742 PMC3285101

[55] Stamatakis A , Jennings J , Ung R et al. A unique population of ventral tegmental area neurons inhibits the lateral habenula to promote reward. Neuron. 2013;80(4):1039‐1053. 24267654 10.1016/j.neuron.2013.08.023 PMC3873746

[56] Zhou L , Liu MZ , Li Q , Deng J , Mu D , Sun YG . Organization of functional long‐range circuits controlling the activity of serotonergic neurons in the dorsal raphe nucleus. Cell Rep. 2017;18(12):3018‐3032. 28329692 10.1016/j.celrep.2017.02.077

[57] Yang Y , Cui Y , Sang K et al. Ketamine blocks bursting in the lateral habenula to rapidly relieve depression. Nature. 2018;554(7692):317‐322. 29446381 10.1038/nature25509

[58] Tian Z , Dong C , Zhang K , Chang L , Hashimoto K . Lack of antidepressant effects of low‐voltage‐sensitive T‐type calcium channel blocker ethosuximide in a chronic social defeat stress model: comparison with (R)‐ketamine. Int J Neuropsychopharmacol. 2018;21(11):1031‐1036. 30085247 10.1093/ijnp/pyy072 PMC6209850

[59] Zhang K , Jia G , Xia L et al. Efficacy of anticonvulsant ethosuximide for major depressive disorder: a randomized, placebo‐control clinical trial. Eur Arch Psychiatry Clin Neurosci. 2020 10.1007/s00406-020-01103-4 32006087

[60] Scheidegger M , Henning A , Walter M et al. Ketamine administration reduces amygdalo‐hippocampal reactivity to emotional stimulation. Hum Brain Mapp. 2016;37(5):1941‐1952. 26915535 10.1002/hbm.23148 PMC6867525

[61] Salvadore G , Cornwell BR , Sambataro F et al. Anterior cingulate desynchronization and functional connectivity with the amygdala during a working memory task predict rapid antidepressant response to ketamine. Neuropsychopharmacology. 2010;35(7):1415‐1422. 20393460 10.1038/npp.2010.24 PMC2869391

[62] Salvadore G , Cornwell BR , Colon‐Rosario V et al. Increased anterior cingulate cortical activity in response to fearful faces: a neurophysiological biomarker that predicts rapid antidepressant response to ketamine. Biol Psychiatry. 2009;65(4):289‐295. 18822408 10.1016/j.biopsych.2008.08.014 PMC2643469

[63] Shirayama Y , Hashimoto K . Effects of a single bilateral infusion of R‐ketamine in the rat brain regions of a learned helplessness model of depression. Eur Arch Psychiatry Clin Neurosci. 2017;267(2):177‐182. 27480092 10.1007/s00406-016-0718-1

[64] Al Shweiki MHDR , Oeckl P , Steinacker P et al. S‐ketamine induces acute changes in the proteome of the mouse amygdala. J Proteomics. 2020;216:103679. 32032757 10.1016/j.jprot.2020.103679

[65] Niesters M , Khalili‐Mahani N , Martini C et al. Effect of subanesthetic ketamine on intrinsic functional brain connectivity: a placebo‐controlled functional magnetic resonance imaging study in healthy male volunteers. Anesthesiology. 2012;117(4):868‐877. 22890117 10.1097/ALN.0b013e31826a0db3

[66] Nissen C , Holz J , Blechert J et al. Learning as a model for neural plasticity in major depression. Biol Psychiatry. 2010;68(6):544‐552. 20655508 10.1016/j.biopsych.2010.05.026

[67] Hogan‐Cann AD , Anderson CM . Physiological roles of non‐neuronal NMDA receptors. Trends Pharmacol Sci. 2016;37(9):750‐767. 27338838 10.1016/j.tips.2016.05.012

[68] Autry AE , Adachi M , Nosyreva E et al. NMDA receptor blockade at rest triggers rapid behavioural antidepressant responses. Nature. 2011;475(7354):91‐95. 21677641 10.1038/nature10130 PMC3172695

[69] Sales AJ , Fogaça MV , Sartim AG et al. Cannabidiol induces rapid and sustained antidepressant‐like effects through increased BDNF signaling and synaptogenesis in the prefrontal cortex. Mol Neurobiol. 2019;56(2):1070‐1081. 29869197 10.1007/s12035-018-1143-4

[70] Lv D , Chen Y , Shen M et al. Mechanisms underlying the rapid‐acting antidepressant‐like effects of neuropeptide VGF (non‐acronymic) C‐terminal peptide TLQP‐62. Neuropharmacology. 2018;143:317‐326. 30291938 10.1016/j.neuropharm.2018.09.046

[71] Kato T , Pothula S , Liu R‐J et al. Sestrin modulator NV‐5138 produces rapid antidepressant effects via direct mTORC1 activation. J Clin Invest. 2019;129(6):2542‐2554. 30990795 10.1172/JCI126859 PMC6546461

[72] Hanania T , Manfredi P , Inturrisi C , Vitolo OV . The N‐methyl‐D‐aspartate receptor antagonist d‐methadone acutely improves depressive‐like behavior in the forced swim test performance of rats. Exp Clin Psychopharmacol. 2019;28(2):196‐201. 10.1037/pha0000310 31368772

[73] Chowdhury GMI , Zhang J , Thomas M et al. Transiently increased glutamate cycling in rat PFC is associated with rapid onset of antidepressant‐like effects. Mol Psychiatry. 2017;22(1):120‐126. 27067013 10.1038/mp.2016.34 PMC5345902

[74] Yellepeddi VK , Zhudeva MY , Movahedi F et al. Biopharmaceutical characterization and oral efficacy of a new rapid acting antidepressant Ro 25–6981. J Pharm Sci. 2018;107(9):2472‐2478. 29800545 10.1016/j.xphs.2018.05.005

[75] Kishimoto T , Chawla JM , Hagi K et al. Single‐dose infusion ketamine and non‐ketamine N‐methyl‐d‐aspartate receptor antagonists for unipolar and bipolar depression: a meta‐analysis of efficacy, safety and time trajectories. Psychol Med. 2016;46(7):1459‐1472. 26867988 10.1017/S0033291716000064 PMC5116384

[76] Rajkumar R , Fam J , Yeo EY , Dawe GS . Ketamine and suicidal ideation in depression: jumping the gun? Pharmacol Res. 2015;99:23‐35. 25982932 10.1016/j.phrs.2015.05.003

[77] Lledo PM , Zhang X , Sudhof TC , Malenka RC , Nicoll RA . Postsynaptic membrane fusion and long‐term potentiation. Science. 1998;279(5349):399‐403. 9430593 10.1126/science.279.5349.399

[78] Huganir RL , Nicoll RA . AMPARs and synaptic plasticity: the last 25 years. Neuron. 2013;80(3):704‐717. 24183021 10.1016/j.neuron.2013.10.025 PMC4195488

[79] Diering GH , Huganir RL . The AMPA receptor code of synaptic plasticity. Neuron. 2018;100(2):314‐329. 30359599 10.1016/j.neuron.2018.10.018 PMC6214363

[80] Kessels HW , Malinow R . Synaptic AMPA receptor plasticity and behavior. Neuron. 2009;61(3):340‐350. 19217372 10.1016/j.neuron.2009.01.015 PMC3917551

[81] Granger AJ , Nicoll RA . Expression mechanisms underlying long‐term potentiation: a postsynaptic view, 10 years on. Philos Trans R Soc Lond B Biol Sci. 2014;369(1633):20130136. 24298139 10.1098/rstb.2013.0136 PMC3843869

[82] Gerhard DM , Pothula S , Liu R‐J et al. GABA interneurons are the cellular trigger for ketamine's rapid antidepressant actions. J Clin Invest. 2020;130:1336‐1349. 31743111 10.1172/JCI130808 PMC7269589

[83] Homayoun H , Moghaddam B . NMDA receptor hypofunction produces opposite effects on prefrontal cortex interneurons and pyramidal neurons. J Neurosci. 2007;27(43):11496‐11500. 17959792 10.1523/JNEUROSCI.2213-07.2007 PMC2954603

[84] Moghaddam B , Adams B , Verma A , Daly D . Activation of glutamatergic neurotransmission by ketamine: a novel step in the pathway from NMDA receptor blockade to dopaminergic and cognitive disruptions associated with the prefrontal cortex. J Neurosci. 1997;17(8):2921‐2927. 9092613 10.1523/JNEUROSCI.17-08-02921.1997 PMC6573099

[85] Xu SX , Zhou ZQ , Li XM , Ji MH , Zhang GF , Yang JJ . The activation of adenosine monophosphate‐activated protein kinase in rat hippocampus contributes to the rapid antidepressant effect of ketamine. Behav Brain Res. 2013;253:305‐309. 23906767 10.1016/j.bbr.2013.07.032

[86] Beurel E , Grieco SF , Amadei C , Downey K , Jope RS . Ketamine‐induced inhibition of glycogen synthase kinase‐3 contributes to the augmentation of alpha‐amino‐3‐hydroxy‐5‐methylisoxazole‐4‐propionic acid (AMPA) receptor signaling. Bipolar Disord. 2016;18(6):473‐480. 27687706 10.1111/bdi.12436 PMC5071181

[87] Liu RJ , Fuchikami M , Dwyer JM , Lepack AE , Duman RS , Aghajanian GK . GSK‐3 inhibition potentiates the synaptogenic and antidepressant‐like effects of subthreshold doses of ketamine. Neuropsychopharmacology. 2013;38(11):2268‐2277. 23680942 10.1038/npp.2013.128 PMC3773678

[88] Cavalleri L , Merlo Pich E , Millan MJ et al. Ketamine enhances structural plasticity in mouse mesencephalic and human iPSC‐derived dopaminergic neurons via AMPAR‐driven BDNF and mTOR signaling. Mol Psychiatry. 2018;23(4):812‐823. 29158584 10.1038/mp.2017.241

[89] Dayas CV , Smith DW , Dunkley PR . An emerging role for the Mammalian target of rapamycin in “pathological” protein translation: relevance to cocaine addiction. Front Pharmacol. 2012;3:13. 22347189 10.3389/fphar.2012.00013 PMC3272624

[90] Duman RS , Monteggia LM . A neurotrophic model for stress‐related mood disorders. Biol Psychiatry. 2006;59(12):1116‐1127. 16631126 10.1016/j.biopsych.2006.02.013

[91] Ihara K , Yoshida H , Jones PB et al. Serum BDNF levels before and after the development of mood disorders: a case‐control study in a population cohort. Transl Psychiatry. 2016;6:e782. 27070410 10.1038/tp.2016.47 PMC4872405

[92] Wu C , Wang Y , He Y et al. Sub‐anesthetic and anesthetic ketamine produce different long‐lasting behavioral phenotypes (24 h post‐treatment) via inducing different brain‐derived neurotrophic factor (BDNF) expression level in the hippocampus. Neurobiol Learn Mem. 2020;167:107136. 31812581 10.1016/j.nlm.2019.107136

[93] Woelfer M , Li M , Colic L et al. Ketamine‐induced changes in plasma brain‐derived neurotrophic factor (BDNF) levels are associated with the resting‐state functional connectivity of the prefrontal cortex. World J Biol Psychiatry. 2019;1‐15. 10.1080/15622975.2019.1679391 31680600

[94] Caffino L , Di Chio M , Giannotti G et al. The modulation of BDNF expression and signalling dissects the antidepressant from the reinforcing properties of ketamine: effects of single infusion vs. chronic self‐administration in rats. Pharmacol Res. 2016;104:22‐30. 26706783 10.1016/j.phrs.2015.12.014

[95] Ninan I , Bath KG , Dagar K et al. The BDNF Val66Met polymorphism impairs NMDA receptor‐dependent synaptic plasticity in the hippocampus. J Neurosci. 2010;30(26):8866‐8870. 20592208 10.1523/JNEUROSCI.1405-10.2010 PMC2911131

[96] Su T‐P , Chen M‐H , Li C‐T et al. Dose‐related effects of adjunctive ketamine in Taiwanese patients with treatment‐resistant depression. Neuropsychopharmacology. 2017;42(13):2482‐2492. 28492279 10.1038/npp.2017.94 PMC5686503

[97] Kowianski P , Lietzau G , Czuba E , Waskow M , Steliga A , Morys J . BDNF: a key factor with multipotent impact on brain signaling and synaptic plasticity. Cell Mol Neurobiol. 2018;38(3):579‐593. 28623429 10.1007/s10571-017-0510-4 PMC5835061

[98] Lu B , Nagappan G , Lu Y . BDNF and synaptic plasticity, cognitive function, and dysfunction. Handb Exp Pharmacol. 2014;220:223‐250. 24668475 10.1007/978-3-642-45106-5_9

[99] Leal G , Bramham CR , Duarte CB . BDNF and hippocampal synaptic plasticity. Vitam Horm. 2017;104:153‐195. 28215294 10.1016/bs.vh.2016.10.004

[100] Jahng JW , Kim JG , Kim HJ , Kim BT , Kang DW , Lee JH . Chronic food restriction in young rats results in depression‐ and anxiety‐like behaviors with decreased expression of serotonin reuptake transporter. Brain Res. 2007;1150:100‐107. 17383614 10.1016/j.brainres.2007.02.080

[101] Golbidi S , Daiber A , Korac B , Li H , Essop MF , Laher I . Health benefits of fasting and caloric restriction. Curr Diab Rep. 2017;17(12):123. 29063418 10.1007/s11892-017-0951-7

[102] Cui R , Fan J , Ge T , Tang L , Li B . The mechanism of acute fasting‐induced antidepressant‐like effects in mice. J Cell Mol Med. 2018;22(1):223‐229. 28782175 10.1111/jcmm.13310 PMC5742683

[103] Ran Y‐H , Hu X‐X , Wang Y‐L et al. YL‐0919, a dual 5‐HT1A partial agonist and SSRI, produces antidepressant‐ and anxiolytic‐like effects in rats subjected to chronic unpredictable stress. Acta Pharmacol Sin. 2018;39(1):12‐23. 28858297 10.1038/aps.2017.83 PMC5758671

[104] Ran Y , Jin Z , Chen X et al. Hypidone hydrochloride (YL‐0919) produces a fast‐onset reversal of the behavioral and synaptic deficits caused by chronic stress exposure. Front Cell Neurosci. 2018;12:395. 30524234 10.3389/fncel.2018.00395 PMC6256289

[105] Li YF . A hypothesis of monoamine (5‐HT) – glutamate/GABA long neural circuit: aiming for fast‐onset antidepressant discovery. Pharmacol Ther. 2020;107494 10.1016/j.pharmthera.2020.107494 31991195

[106] Ji MJ , Zhang XY , Chen Z , Wang JJ , Zhu JN . Orexin prevents depressive‐like behavior by promoting stress resilience. Mol Psychiatry. 2019;24(2):282‐293. 30087452 10.1038/s41380-018-0127-0 PMC6755988

[107] Lutter M , Krishnan V , Russo SJ , Jung S , McClung CA , Nestler EJ . Orexin signaling mediates the antidepressant‐like effect of calorie restriction. J Neurosci. 2008;28(12):3071‐3075. 18354010 10.1523/JNEUROSCI.5584-07.2008 PMC2713756

[108] Yang L , Zou B , Xiong X et al. Hypocretin/orexin neurons contribute to hippocampus‐dependent social memory and synaptic plasticity in mice. J Neurosci. 2013;33(12):5275‐5284. 23516292 10.1523/JNEUROSCI.3200-12.2013 PMC3640412

[109] Lu GL , Lee CH , Chiou LC . Orexin A induces bidirectional modulation of synaptic plasticity: inhibiting long‐term potentiation and preventing depotentiation. Neuropharmacology. 2016;107:168‐180. 26965217 10.1016/j.neuropharm.2016.03.005

[110] Huang CC , Chou D , Yeh CM , Hsu KS . Acute food deprivation enhances fear extinction but inhibits long‐term depression in the lateral amygdala via ghrelin signaling. Neuropharmacology. 2016;101:36‐45. 26384653 10.1016/j.neuropharm.2015.09.018

[111] Lu Y , Niu M , Qiu X et al. Acute but not chronic calorie restriction defends against stress‐related anxiety and despair in a GHS‐R1a‐dependent manner. Neuroscience. 2019;412:94‐104. 31185255 10.1016/j.neuroscience.2019.05.067

[112] Huang H‐J , Chen X‐R , Han Q‐Q et al. The protective effects of Ghrelin/GHSR on hippocampal neurogenesis in CUMS mice. Neuropharmacology. 2019;155:31‐43. 31103617 10.1016/j.neuropharm.2019.05.013

[113] Huang H‐J , Zhu X‐C , Han Q‐Q et al. Ghrelin alleviates anxiety‐ and depression‐like behaviors induced by chronic unpredictable mild stress in rodents. Behav Brain Res. 2017;326:33‐43. 28245976 10.1016/j.bbr.2017.02.040

[114] Eslami M , Sadeghi B , Goshadrou F . Chronic ghrelin administration restores hippocampal long‐term potentiation and ameliorates memory impairment in rat model of Alzheimer's disease. Hippocampus. 2018;28(10):724‐734. 30009391 10.1002/hipo.23002

[115] Ribeiro LF , Catarino T , Santos SD et al. Ghrelin triggers the synaptic incorporation of AMPA receptors in the hippocampus. Proc Natl Acad Sci USA. 2014;111(1):E149‐E158. 24367106 10.1073/pnas.1313798111 PMC3890894

[116] Chen L , Xing T , Wang M et al. Local infusion of ghrelin enhanced hippocampal synaptic plasticity and spatial memory through activation of phosphoinositide 3‐kinase in the dentate gyrus of adult rats. Eur J Neuorsci. 2011;33(2):266‐275. 10.1111/j.1460-9568.2010.07491.x 21219473

[117] Cavalier M , Crouzin N , Ben Sedrine A et al. Involvement of PKA and ERK pathways in ghrelin‐induced long‐lasting potentiation of excitatory synaptic transmission in the CA1 area of rat hippocampus. Eur J Neuorsci. 2015;42(8):2568‐2576. 10.1111/ejn.13013 26153524

[118] Ghosal S , Bang E , Yue W et al. Activity‐dependent brain‐derived neurotrophic factor release is required for the rapid antidepressant actions of scopolamine. Biol Psychiatry. 2018;83(1):29‐37. 28751069 10.1016/j.biopsych.2017.06.017 PMC5705490

[119] Wohleb ES , Wu M , Gerhard DM et al. GABA interneurons mediate the rapid antidepressant‐like effects of scopolamine. J Clin Invest. 2016;126(7):2482‐2494. 27270172 10.1172/JCI85033 PMC4922686

[120] Tsai H‐H , Li H , Fuentealba LC et al. Regional astrocyte allocation regulates CNS synaptogenesis and repair. Science. 2012;337(6092):358‐362. 22745251 10.1126/science.1222381 PMC4059181

[121] Wang Y , Xie L , Gao C , Zhai L , Zhang N , Guo L . Astrocytes activation contributes to the antidepressant‐like effect of ketamine but not scopolamine. Pharmacol Biochem Behav. 2018;170:1‐8. 29729289 10.1016/j.pbb.2018.05.001

[122] Ardalan M , Rafati AH , Nyengaard JR , Wegener G . Rapid antidepressant effect of ketamine correlates with astroglial plasticity in the hippocampus. Br J Pharmacol. 2017;174(6):483‐492. 28087979 10.1111/bph.13714 PMC5323512

[123] Mishra PK , Kumar A , Behar KL , Patel AB . Subanesthetic ketamine reverses neuronal and astroglial metabolic activity deficits in a social defeat model of depression. J Neurochem. 2018;146(6):722‐734. 29964293 10.1111/jnc.14544

[124] Kim YK , Na KS . Role of glutamate receptors and glial cells in the pathophysiology of treatment‐resistant depression. Prog Neuropsychopharmacol Biol Psychiatry. 2016;70:117‐126. 27046518 10.1016/j.pnpbp.2016.03.009

[125] Chang Y , Lee JJ , Hsieh CY , Hsiao G , Chou DS , Sheu JR . Inhibitory effects of ketamine on lipopolysaccharide‐induced microglial activation. Mediators Inflamm. 2009;2009:705379. 19343193 10.1155/2009/705379 PMC2662525

[126] Ho MF , Zhang C , Zhang L , Li H , Weinshilboum RM . Ketamine and active ketamine metabolites regulate STAT3 and the type I interferon pathway in human microglia: molecular mechanisms linked to the antidepressant effects of ketamine. Front Pharmacol. 2019;10:1302. 31827434 10.3389/fphar.2019.01302 PMC6848891

[127] Steiner J , Walter M , Gos T et al. Severe depression is associated with increased microglial quinolinic acid in subregions of the anterior cingulate gyrus: evidence for an immune‐modulated glutamatergic neurotransmission? J Neuroinflammation. 2011;8:94. 21831269 10.1186/1742-2094-8-94 PMC3177898

[128] Guillemin GJ , Smythe G , Takikawa O , Brew BJ . Expression of indoleamine 2,3‐dioxygenase and production of quinolinic acid by human microglia, astrocytes, and neurons. Glia. 2005;49(1):15‐23. 15390107 10.1002/glia.20090

[129] Verdonk F , Petit A‐C , Abdel‐Ahad P et al. Microglial production of quinolinic acid as a target and a biomarker of the antidepressant effect of ketamine. Brain Behav Immun. 2019;81:361‐373. 31255681 10.1016/j.bbi.2019.06.033

[130] Wohleb ES , Franklin T , Iwata M , Duman RS . Integrating neuroimmune systems in the neurobiology of depression. Nat Rev Neurosci. 2016;17(8):497‐511. 27277867 10.1038/nrn.2016.69

[131] Xu Y , Sheng H , Bao Q , Wang Y , Lu J , Ni X . NLRP3 inflammasome activation mediates estrogen deficiency‐induced depression‐ and anxiety‐like behavior and hippocampal inflammation in mice. Brain Behav Immun. 2016;56:175‐186. 26928197 10.1016/j.bbi.2016.02.022

[132] Kubera M , Obuchowicz E , Goehler L , Brzeszcz J , Maes M . In animal models, psychosocial stress‐induced (neuro)inflammation, apoptosis and reduced neurogenesis are associated to the onset of depression. Prog Neuropsychopharmacol Biol Psychiatry. 2011;35(3):744‐759. 20828592 10.1016/j.pnpbp.2010.08.026

[133] Ironside M , Admon R , Maddox SA et al. Inflammation and depressive phenotypes: evidence from medical records from over 12 000 patients and brain morphology. Psychol Med. 2019;1–9. 10.1017/s0033291719002940 PMC7160032 31615590

[134] Tan S , Wang Y , Chen K , Long Z , Zou J . Ketamine alleviates depressive‐like behaviors via down‐regulating inflammatory cytokines induced by chronic restraint stress in mice. Biol Pharm Bull. 2017;40(8):1260‐1267. 28769008 10.1248/bpb.b17-00131

[135] Clarke M , Razmjou S , Prowse N et al. Ketamine modulates hippocampal neurogenesis and pro‐inflammatory cytokines but not stressor induced neurochemical changes. Neuropharmacology. 2017;112(Pt A):210‐220. 27106168 10.1016/j.neuropharm.2016.04.021

[136] Nowak W , Grendas LN , Sanmarco LM et al. Pro‐inflammatory monocyte profile in patients with major depressive disorder and suicide behaviour and how ketamine induces anti‐inflammatory M2 macrophages by NMDAR and mTOR. EBioMedicine. 2019;50:290‐305. 31753725 10.1016/j.ebiom.2019.10.063 PMC6921226

[137] Zhang J‐C , Yao W , Dong C et al. Blockade of interleukin‐6 receptor in the periphery promotes rapid and sustained antidepressant actions: a possible role of gut‐microbiota‐brain axis. Transl Psychiatry. 2017;7(5):e1138. 28556833 10.1038/tp.2017.112 PMC5534942

[138] Yang JJ , Wang N , Yang C , Shi JY , Yu HY , Hashimoto K . Serum interleukin‐6 is a predictive biomarker for ketamine's antidepressant effect in treatment‐resistant patients with major depression. Biol Psychiatry. 2015;77(3):e19‐e20. 25104172 10.1016/j.biopsych.2014.06.021

[139] Yang C , Wardenaar KJ , Bosker FJ , Li J , Schoevers RA . Inflammatory markers and treatment outcome in treatment resistant depression: a systematic review. J Affect Disord. 2019;257:640‐649. 31357161 10.1016/j.jad.2019.07.045

[140] Wu JC , Bunney WE . The biological basis of an antidepressant response to sleep deprivation and relapse: review and hypothesis. Am J Psychiatry. 1990;147(1):14‐21. 2403471 10.1176/ajp.147.1.14

[141] Lopez‐Rodriguez F , Kim J , Poland RE . Total sleep deprivation decreases immobility in the forced‐swim test. Neuropsychopharmacology. 2004;29(6):1105‐1111. 14970835 10.1038/sj.npp.1300406

[142] Hines DJ , Schmitt LI , Hines RM , Moss SJ , Haydon PG . Antidepressant effects of sleep deprivation require astrocyte‐dependent adenosine mediated signaling. Transl Psychiatry. 2013;3:e212. 23321809 10.1038/tp.2012.136 PMC3566717

[143] Serchov T , Clement H‐W , Schwarz M et al. Increased signaling via adenosine A1 receptors, sleep deprivation, imipramine, and ketamine inhibit depressive‐like behavior via induction of Homer1a. Neuron. 2015;87(3):549‐562. 26247862 10.1016/j.neuron.2015.07.010 PMC4803038

[144] Serchov T , Schwarz I , Theiss A et al. Enhanced adenosine A1 receptor and Homer1a expression in hippocampus modulates the resilience to stress‐induced depression‐like behavior. Neuropharmacology. 2020;162:107834. 31682853 10.1016/j.neuropharm.2019.107834

[145] Holz A , Mülsch F , Schwarz MK et al. Enhanced mGlu5 signaling in excitatory neurons promotes rapid antidepressant effects via AMPA receptor activation. Neuron. 2019;104(2):338‐352.e337. 31420117 10.1016/j.neuron.2019.07.011

[146] Vyazovskiy VV , Cirelli C , Pfister‐Genskow M , Faraguna U , Tononi G . Molecular and electrophysiological evidence for net synaptic potentiation in wake and depression in sleep. Nat Neurosci. 2008;11(2):200‐208. 18204445 10.1038/nn2035

[147] Tononi G , Cirelli C . Sleep and the price of plasticity: from synaptic and cellular homeostasis to memory consolidation and integration. Neuron. 2014;81(1):12‐34. 24411729 10.1016/j.neuron.2013.12.025 PMC3921176

[148] Wisor JP , Pasumarthi RK , Gerashchenko D et al. Sleep deprivation effects on circadian clock gene expression in the cerebral cortex parallel electroencephalographic differences among mouse strains. J Neurosci. 2008;28(28):7193‐7201. 18614689 10.1523/JNEUROSCI.1150-08.2008 PMC2603080

[149] Orozco‐Solis R , Montellier E , Aguilar‐Arnal L et al. A circadian genomic signature common to ketamine and sleep deprivation in the anterior cingulate cortex. Biol Psychiatry. 2017;82(5):351‐360. 28395871 10.1016/j.biopsych.2017.02.1176 PMC5660920

[150] Bunney BG , Li JZ , Walsh DM et al. Circadian dysregulation of clock genes: clues to rapid treatments in major depressive disorder. Mol Psychiatry. 2015;20(1):48‐55. 25349171 10.1038/mp.2014.138 PMC4765913

[151] van Calker D , Biber K , Domschke K , Serchov T . The role of adenosine receptors in mood and anxiety disorders. J Neurochem. 2019;151(1):11‐27. 31361031 10.1111/jnc.14841

[152] Podkowa K , Pochwat B , Brański P , Pilc A , Pałucha-Poniewiera A . Group II mGlu receptor antagonist LY341495 enhances the antidepressant-like effects of ketamine in the forced swim test in rats. Psychopharmacology. 2016;233(15-16):2901-2914. 10.1007/s00213-016-4325-7 27286960 PMC4933730

[153] Petryshen TL , Lewis MC , Dennehy KA , Garza JC , Fava M . Antidepressant-like effect of low dose ketamine and scopolamine co-treatment in mice. Neuroscience Letters. 2016;620:70-73. 10.1016/j.neulet.2016.03.051 27033002

[154] Kordjazy N , Haj-Mirzaian A , Amiri S , Ostadhadi S , Amini-khoei H , Dehpour AR . Involvement of N-methyl-d-aspartate receptors in the antidepressant-like effect of 5-hydroxytryptamine 3 antagonists in mouse forced swimming test and tail suspension test. Pharmacology Biochemistry and Behavior. 2016;141:1-9. 10.1016/j.pbb.2015.11.009 26604075

